# Activating KIRs in Chronic Lymphoproliferative Disorder of NK Cells: Protection from Viruses and Disease Induction?

**DOI:** 10.3389/fimmu.2014.00072

**Published:** 2014-02-26

**Authors:** Renato Zambello, Antonella Teramo, Gregorio Barilà, Cristina Gattazzo, Gianpietro Semenzato

**Affiliations:** ^1^Hematology and Clinical Immunology Branch, Department of Medicine, Padua University School of Medicine, Padua, Italy; ^2^Venetian Institute of Molecular Medicine, Padua, Italy

**Keywords:** CLPD-NK, activating KIRs, viral infections, NK cell licensing, pathogenesis in NK disorders

## Abstract

Human natural killer (NK) cells are functionally regulated by killer cell immunoglobulin-like receptors (KIRs) and their interactions with HLA class I molecules. As KIR expression in a given NK cell is stochastically established, KIR repertoire perturbations reflect a dominance of discrete NK-cell subsets as the consequence of adaptation of the NK-cell compartment to exogenous agents, more often represented by virus infection. Although inhibitory interactions between KIR and their cognate HLA class I ligands abrogate effector responses of NK cells, they are also required for the functional education of NK cell. The biology and molecular specificities of the activating KIRs are less well defined, and most interactions with presumed HLA class I ligands are weak. Interestingly, epidemiologic studies link activating KIR genes to resistance against numerous virus infections. Chronic lymphoproliferative disorder of NK cells (CLPD-NK) is an indolent NK cell disease characterized by a persistent increase of circulating NK cells (usually exceeding 500 NK cells/mm^3^). The mechanism through which NK cells are induced to proliferate during CLPD-NK pathogenesis is still a matter of debate. Accumulating data suggest that exogenous agents, in particular viruses, might play a role. The etiology of CLPD-NK, however, is largely unknown. This is likely due to the fact that not a single, specific agent is responsible for the NK cells proliferation, which perhaps represents the expression of an abnormal processing of different foreign antigens, sharing a chronic inflammatory background. Interestingly, proliferating NK cells are typically characterized by expression of a restricted pattern of KIR, which have been demonstrated to be mostly represented by the activating form. This finding indicates that these receptors may be directly involved in the priming of NK cells proliferation.

## Introduction

**Figure 1:**
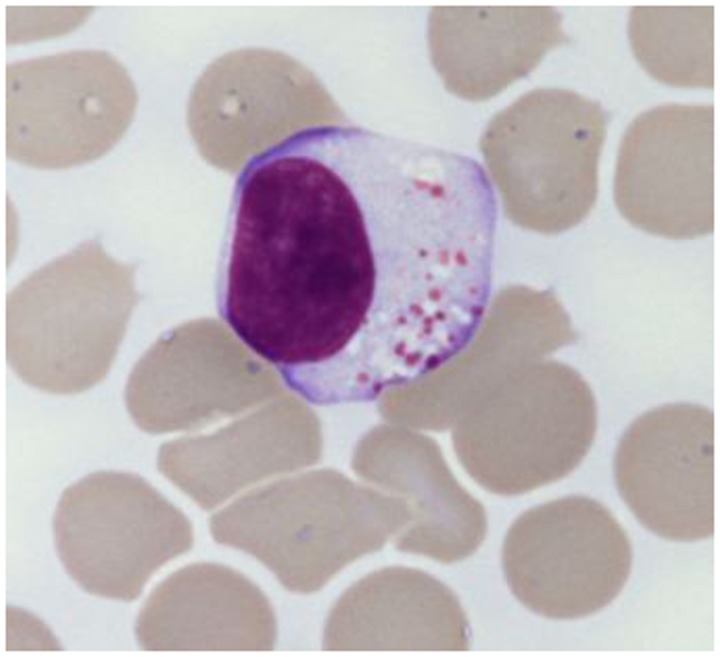
**Typical large granular lymphocyte morphology as detected in a CLPD-NK patient**.

The chronic lymphoproliferative disorders of NK cells (CLPD-NK) are included among the novelties of the current World Health Organization (WHO) classification (1). These rare and heterogeneous disorders are characterized by a chronic expansion of mature appearing NK cells (usually more than 500/μl) in peripheral blood for more than 6 months (2–6), without a clearly identified cause (Figure 1). Patients are usually adults with a mean age of 60 years without gender and racial predisposition (7). The circulating cells show typical large granular lymphocyte (LGL) morphology, with moderate amount of pale cytoplasm that contains ≥3 azurophilic granules. Bone marrow biopsy is characterized by interstitial infiltration of cells with small nuclei and pale cytoplasm, which are difficult to recognize without the help of immunohistochemical techniques. Pathological NK cells express CD16 and usually low levels of CD56 and CD57. As expected, cells express T-cell intracellular antigen 1 (TIA1), granzyme and perforins, which correlate with the cytotoxic potential of these cells displayed *in vitro* cytotoxic assays. CD94 antigen is expressed at high density on patients’ NK cells; this antigen is usually associated with the inhibitory subunit NKG2A, although in a relevant number of cases the association CD94/NKG2C has been reported (8). Patients’ NK cells characteristically express functional β and γ chains of IL-2/IL-15 receptor, which are strictly related to the role of these cytokines in the pathogenesis of disease (9). Cytogenetic is normal in most cases (9) and the germ line configuration of TCR is usually demonstrated, as expected for normal NK cells. Since clonality of proliferating cells is difficult to detect in these patients, the analysis of restriction fragment length polymorphism (RFLP) has been used as marker to demonstrate the clonality in some but not all patients. The evidence of a restricted pattern of clonally distributed KIR genes expression by proliferating NK cells might also provide indirect demonstration of clonality (10). In rare case in which EBV can be demonstrated in plasmid form within NK cells, the clonality of cells might be easily examined by Southern Blot analysis using probes recognizing the EBV terminal repeats (11).

Most patients are asymptomatic, and the disease has a chronic indolent clinical course, similar to that reported for patients with T-LGL leukemia (T-LGLL) (2–6); in these cases, the disease is diagnosed by blood analysis performed for other purposes. In some cases, this disorder is associated with other conditions, including pure red cell aplasia, vasculitic syndromes, solid and hematologic tumors, splenectomy, neuropathy, and autoimmune disorders (2–6). Recently in patients with chronic myelogenous leukemia, the association has been reported between treatment with dasatinib and the development of CLPD-NK. It has been suggested that the development of CLPD-NK might have a therapeutic effect on Ph positive leukemic cells (12). Systemic symptoms, such as cytopenia (mostly neutropenia and anemia), are uncommon. Lymphadenopathy, hepatomegaly, splenomegaly, and cutaneous lesions are uncommon. Occasionally, patients present with a slowly progressive increase of peripheral blood NK cells and with organ involvement. In rare cases, the disease transforms to aggressive NK cell leukemia (13). These EBV positive patients usually suffer from chronic active EBV infection and should carefully be monitored for the emergence of clonal cells (11). Several cases with a spontaneous complete remission have been reported (14). Patients with CLPD-NK usually have an indolent clinical course and respond to immunosuppressive therapy with low doses of methotrexate (usually 10 mg/m^2^/week) or cyclophosphamide (50 or 100 mg/day) or cyclosporin (3–5 mg/kg/day) with or without inclusion of low doses of steroid (15). Because of the potential long-term side effects of immunosuppressive therapy, limiting specific therapy only to patients with symptomatic disease is recommended.

## CLPD-NK Express Activating KIR

In recent years, several studies have been published focusing on the pathogenetic mechanisms of this disease (9, 16–20). A genetic susceptibility for this disease has been suggested and has been related to the detection in these patients of type B *KIR* gene repertoire, which is characterized by a high number of activating genes (21). As indicated above, a restricted pattern of KIR expression has usually been reported in these patients, which is characterized either by a dominant expression of a relevant KIR, or by a lack of KIR expression (22). A typical feature of these patients is the preferential expression of the KIR activating receptors isoforms (10, 23). Together with a bias toward activating KIR expression, a deep silencing of inhibitory KIR through increased gene methylation has been demonstrated by our group (19). More specifically, we showed the complete lack of KIR3DL1 expression in most analyzed patients, being the receptor expressed in 13% of patients as compared to 90% of controls (*p* < 0.01). Interestingly, the results of methylation patterns of *KIR3DL1* promoter showed a significantly higher methylation status (0.76 ± 0.12 SD) in the patients with respect to the healthy subjects (0.49 ± 0.10 SD, *p* < 0.01). These data suggest that together with the increased expression of activating receptors, the lack of the inhibitory signal could also play a role in the pathogenesis of disease (19). Only few studies addressed the expression of NCR, NKG2D, and other activating receptors. We investigated the expression of these receptors in a series of 18 cases of LDGL patients and showed that, among NCR antigen expression, NKp30 was strongly down regulated in all but one of the cases analyzed. Similarly the NKp46 receptor in most instances was detected only in small fractions of NK cells (10). These peculiar phenotypic results in these patients suggested the occurrence of a defect in NCR expression that was reminiscent of that reported in acute myeloid leukemia patients (24). Regarding NKp44, which is normally expressed only in activated NK cells, this receptor was not expressed at significant levels on LGL surface of the patients analyzed. A completely different pattern was observed for NKG2D, NKp80, and 2B4 molecules that were homogeneously present on NK cells in the majority of the patients. All together, these data indicate that in most cases patients’ NK cells express normal levels of NKG2D while NCR molecules are generally present at low density. In addition, although in most patients NK cells were characterized by the CD94/NKG2A+ phenotype, a minor fraction of cases (nearly 20%) expressed CD94+/NKG2C+ phenotype (10).

## CLPD-NK and Viral Infections

**Figure 2:**
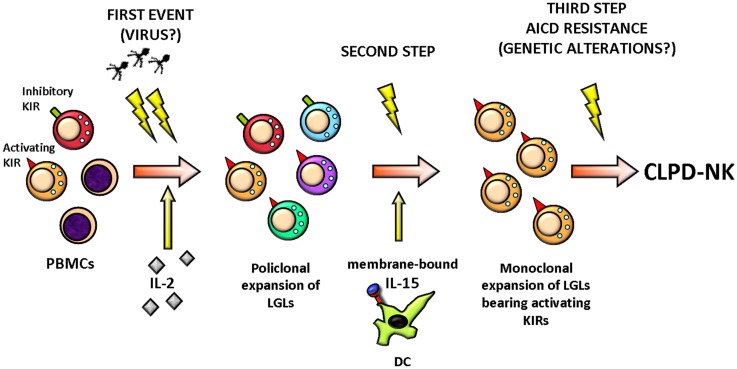
**Hypothetical pathogenic events leading to CLPD-NK**. A stimulation by exogenous antigen(s), such as virus, represents the initial stimulus inducing the activation and clonal expansion of NK cells, selecting preferentially NK cells equipped with activating KIR, probably because these NK cells are more responsive. LGL expansion at the beginning could be polyclonal but then it becomes monoclonal and chronic, sustained by the action of cytokines, like IL-2 and IL-15. Finally, the clonal LGL expansion does not encounter a resolution for a third event establishing the activation induced cell death (AICD) resistance, probably due to a genetic alteration. All these events result in LGL aberrant outliving and number increase. PBMCs, peripheral blood mononuclear cells; KIR, killer immunoglobulin-like receptor; LGLs, large granular lymphocytes; DC, dendritic cell; AICD, activation induced cell death; CLPD-NK, chronic lymphoproliferative disease of natural killer cells.

Natural killer cell activation in response to an unknown stimulus, likely of viral origin, is postulated to play a role in the initial steps of CLPD-NK by selecting NK clones (Figure 2) (25). Although no prototypical HTLV infection was demonstrated in these patients, the evidence that in 73% of cases sera from a series of patients from Europe and USA reacted with the recombinant HTLV env protein p21E suggests that exposure to a protein containing homology to BA21 may be important in the pathogenesis of this lymphoproliferative disorder (16, 26).

In contrast with other mature NK cell neoplasms, EBV DNA is not usually detected within affected lymphocytes in USA and Europe countries (27) (6/16 NK LGL positive for EBV DNA), whereas a significative high incidence has been reported in Japanese patients, usually correlating with a more aggressive clinical behavior (11). Anyway the link between EBV infection and LGL disease is sustained by the observation that spontaneous resolution of KIR restricted LGL associated with disappearance of EBV DNA (28). Among Herpes viruses, Human CMV has been reported to be crucial in influencing NK receptor expression in NK cells (29). Remarkably, elevated numbers of CD94/NKG2C+ NK cells, previously shown to expand in association to CMV infection (30), were preferentially found in Vbeta13.1+ CD4+ T-LGLL, further supporting its role in the pathogenesis of a subset of T-LGLL (31).

It is believed that bone marrow, which is frequently involved in CLPD-NK patients, represents the setting where the putative inciting antigen could reside and dendritic cells (DCs) have been suggested to represent the target of infection in these patients (17). Interestingly, analysis of bone marrow biopsies of patients demonstrated a topographic distribution of DCs and NK cells that indicates a close contact between the two cell types (17). DCs are also likely to represent the source of IL-15, which is crucial in the mechanisms sustaining the maintenance of NK proliferation. IL-15 has been found to mediate its activity by altering Bcl-2 family members, and more specifically by modulating Bid expression (18).

## Activating KIR and Viral Protection: Is it True in CLPD-NK?

Epidemiologic studies link activating KIR genes to resistance against numerous virus infections (32). Beziat et al. showed that infection with human CMV induce expansion and differentiation of KIR-expressing NK cells, causing as stable imprints in the repertoire (33). Interestingly, these authors showed that NK education by inhibitory killer cell immunoglobulin-like receptors (KIRs) was associated with a unique contribution of activating KIRs (KIR2DS4, KIR2DS2, or KIR3DS1), in addition to NKG2C, in the expansion of human NK cells. Interestingly, CMV-associated factors have been suggested to specifically influence KIR gene expression by regulating epigenetic expression of KIR genes (34). In addition, in allogeneic bone marrow transplantation setting, donor KIR2DS1 has been reported to protect against human CMV reactivation (35). KIR3DS1 in conjunction with HLA-Bw4 with an isoleucine at position 80 has been reported to be associated with slower progression of HIV infection to AIDS (36). Pelac et al. demonstrated that NK cells from individuals with multiple copies of KIR3DL1, in the presence of KIR3DS1 and the appropriate ligands inhibit HIV-1 replication (37). This is frequently associated with a significant expansion of KIR3DS1+, but not KIR3DL1+, NK cells in HIV positive patients’ peripheral blood. Epidemiological studies have indicated a protective effect for the activating *KIR2DS1* and *KIR3DS1 genes* also in patients with EBV-associated Hodgkin’s lymphoma, although there is as yet no direct evidence for the involvement of these receptors in the recognition of EBV-transformed cells (38). *KIR3DS1* genotype has been shown to exert a positive effect on HCV viral clearance during the first weeks of treatment in HCV/HIV infected patients (39).

Although many of the above reported viral infections are at least in part controlled by activating KIRs, a direct proof of the role of these NK receptors in controlling a putative viral infection in patients with CLPD is not available. This is likely due to the fact that not a single, specific agent is responsible for the NK proliferation, which perhaps represents the expression of an abnormal processing of different foreign antigens.

## Are CLPD-NK Cells Licensed?

It is well known that NK functions are regulated by the integration of signals received from activating and inhibitory receptors. To prevent inadvertent activation against normal tissues, NK must be educated to tolerate self, this process, termed “licensing,” acting through an MHC-dependent mechanism requiring interaction between inhibitory KIR and cognate MHC class I ligands (40). Up to date, two models have been proposed to describe NK cell education (41): “arming” model and “disarming” model. In “arming” model, which is the best-known model proposed by Raulet and Vance (42), NK cells acquire functional competence after ligation of inhibitory receptors by self MHC class I molecules, while NK cells lacking these inhibitory receptors for self MHC enter in an “anergy” state. In this way, engagement of inhibitory receptor by self MHC during NK cell maturation guarantees NK cells to acquire “license to kill” and to become fully functional competent.

Natural killer licensing involves not only inhibitory KIRs but also activating KIRs have probably an underestimated role in this process, which is complementary but with different outcome, since it has been reported that education via activating KIRs decreases NK cells response (43). However, “unlicensed” NK are not so “anergic” but seem to have a crucial role in discrete settings. As an example, in murine model, unlicensed NK cells were the main mediators of NK cell-mediated control of mouse cytomegalovirus infection *in vivo*. In fact, depletion of unlicensed NK cells impaired control of viral titers, but depletion of licensed NK cells did not. Furthermore, the transfer of unlicensed NK cells was more protective than the transfer of licensed NK cells, indicating that unlicensed NK cells are critical for protection against viral infection (44).

As stated above, in CLPD-NK, proliferating NK cells are characterized by skewed KIR expression and selection and expansion of NK subset expressing activating KIRs, instead of the more common NK cells expressing inhibitory KIRs, represent a crucial step in the development of this disorder (10). Based on the recent insights in NK cell education and function, in CLPD-NK, we can suppose that expansion of long-lived NK cells involves “unlicensed” NK equipped with activating KIRs, which have a predominant role during viral infections, in accordance with the hypothesis that viral stimulation may be the starting trigger of the disorder. According with this suggestion, we performed HLA genotyping analysis in 29 CLPD-NK patients showing that in 93% of cases, a KIR/HLA-I mismatch was present indicating that NK cell proliferation in CLPD is mostly represented by unlicensed cells (21). Considering that in healthy individuals, KIR/HLA-I genetic mismatch has been detected in nearly 50% of cases, our results point to a role of the KIR/HLA-I mismatch in the pathogenesis of the disease.

## Conclusive Remarks

Chronic lymphoproliferative disorders of NK cells are characterized by the expression of a restricted pattern of activating KIR receptors on proliferating NK cells. It is believed from indirect evidence that exogenous agent(s), likely of viral origin, might contribute to the initial steps of disease. Recent knowledge on the mechanisms of NK cell function and different contribution of NK cell receptors against viral infections might help in the comprehension of the mechanisms leading to selection of a discrete NK cell population. A possible inciting role of the putative antigen within DCs in the bone marrow can be suggested. The inability of antigen clearance and/or the occurrence of new events might contribute to the persistence of NK clone. In this way, the identification that STAT3 SH2 somatic mutations (45, 46), which can be found in a fraction of CLPD-NK patients, might indicate that an acquired genetic mutation could contribute to the immortalization of NK proliferation.

## Conflict of Interest Statement

The authors declare that the research was conducted in the absence of any commercial or financial relationships that could be construed as a potential conflict of interest.
